# The use of simulation as a teaching modality for paramedic education: a scoping review

**DOI:** 10.29045/14784726.2020.12.5.3.31

**Published:** 2020-12-01

**Authors:** Bethany Wheeler, Enrico Dippenaar

**Affiliations:** Emergency Medicine Research Group, Anglia Ruskin University; Emergency Medicine Research Group, Anglia Ruskin University ORCID ID: https://orcid.org/0000-0001-8406-7373

**Keywords:** education, paramedic, simulation, teaching

## Abstract

**Background:**

Simulation is a broad concept used as an education pedagogy for a wide range of disciplines. The use of simulation to educate paramedics is a frequently used but untested modality to teach psycho-motor skills, acquire new knowledge and gain competence in practice. This review identifies how simulation is currently being used for the education of paramedics, and establish the context for future application.

**Methods:**

A scoping review of the literature was undertaken following the PRISMA systematic approach. Flexible inclusion criteria were used to capture research and non-research articles that would contribute to the synthesis of literature with a specific knowledge base pertaining to simulation use for paramedic education.

**Results:**

Initial searching yielded 1388 records, of which 22 remained after initial title and abstract reading. Following secondary full-text screening, 18 articles were deemed appropriate for final inclusion: eight are research, two literature reviews and eight non-research. Across all the literature, a range of concepts are discussed: Skill vs Scenario, Virtual Learning, Inter-Professional Learning, Fidelity, Cost, Equipment, Improvement of Competency, Patient Safety, Perception of Simulation.

**Conclusion:**

It is evident that simulation is a primary teaching modality, consistently used to educate and train paramedics. Simulation is inherently effective at teaching clinical skills and building student competence in particular areas. Similarly, simulation is effective at providing paramedics with experiences and opportunities to learn in varied environments using differing techniques. This allows students to apply the relevant skills and knowledge when faced with real patients.

## Background

Simulation is a broad concept used as an educational pedagogy for a wide range of disciplines, though educational theorists debate what constitutes it. The most accepted definition is:

An array of structured activities that represent actual or potential situations in education and practice. These activities allow participants to develop or enhance their knowledge, skills, and attitudes, or to analyze and respond to realistic situations in a simulated environment. (Lopreiato et al., 2020)

The use of Simulation-Based Education (SBE) to educate paramedics is a well-established training modality that stems from the armed forces (Bradley, 2006; Stamper et al., 2008). Regardless of pathway through to registration, student paramedics spend a substantial portion of their education undergoing simulation-based training (NHTSA, 2009). These simulations have a variety of intended outcomes, dependent on the skills or lessons taught. At a lower cognitive level, simulation is used to develop simple psycho-motor skills to gain competence with a procedure or technique (Abdulmohsen, 2010; Dent, 2001). At a higher cognitive level, it is used to challenge the student’s ability to problem-solve and adapt to the patient presented (Cannon-Bowers, 2008; Cheng et al., 2007; Ziv et al., 2005).

The definition of simulation modality is vague, and may be construed differently between individuals. Simulation modality is an umbrella term meaning *the type of simulation being used as part of the simulation activity* (Lopreiato et al., 2020). This leaves room for individual interpretation; use of a skills trainer such as an airway manikin may constitute simulation to some learners, while others would require a clinical context and setting to meet their expectations (Chiniara et al., 2013; Hassan and Sloan, 2006; Kobayashi et al., 2006). This is dependent upon the learner’s own experience and competence. Does classical, step-by-step imitation by a learner mimicking a tutor qualify as simulation? Frasson and Blanchard (2012) state that the evolution of simulation has changed its definition, being no longer only associated with computers but specific to each discipline.

SBE in paramedic education provides registered and non-registered clinicians with real-life presentations. The paramedic profession incorporates a wide range of clinicians, from undergraduate students and newly qualified paramedics to specialist and advanced paramedics (College of Paramedics, 2014). The educational needs of learners evolve with experience and time, with the provided simulation designed to meet these needs. A scoping review was conducted to establish how simulation is currently being utilised for paramedic education, and the context for future application.

## Methods

A scoping review of the literature was undertaken between February and April 2019. A scoping review provides broad coverage of the body of literature in the area of interest, summarising the evidence (Levac, 2010; Munn et al., 2018; Peters, 2015). This methodology was chosen to allow for a broad range of literature (research and non-research) to be included and to highlight key concepts and gaps in knowledge, and provide sources of evidence that could inform current practices. Given the paucity of literature specific to paramedic simulation-based education, the intention of this review is to capture as much literature as possible to map and highlight what has been published on this topic.

The scoping review was conducted using the PRISMA (Preferred Reporting Items for Systematic Reviews and Meta-Analyses) systematic approach, but modified for a broader inclusion criteria than a traditional systematic review. The search was limited to English language publications that had full text available, including those requiring institutional access. This may have introduced bias, however it allowed for more literature to be covered and included in the summary of evidence. The Boolean search key words are presented in Table 1. The search was non-specific in the type of literature searched, thus including research and non-research papers. It was conducted across seven different databases, primarily medical and educational, as shown in Table 2. Due to the nature of the review, the databases were selected for comprehensiveness and range of literature available.

**Table 1.** Keywords employed in the literature search.

Group 1	Group 2	Group 3	Group 4
**Simulation**	**Modality**	**Paramedic**	**Education**
reproduce* scenario simulate* ‘simulation-based’	effect* method process	ambulance ‘ambulance personnel’ ‘ambulance staff’ clinician ‘emergency care’ ‘emergency medical’ ‘EMS staff’ EMT practitioner technician	learn* student study* teach* train*

Words within and between groups were combined with AND/OR. ‘Phrase searching’; *Truncation.

**Table 2.** The selection process.

Databases	Hit number	Included^a^	Research	Non-research
	**1388**	**18**	**10**	**8**
*Medicine* ARU library ^b^ Cochrane PubMed ScienceDirect	141 18 627 566	6 1 6 4	1 1 5 2	5 1 2
*Educational* British Education Index Professional Development Collection	15 21	1	1	

^a^ Inclusion is based on full-text availability, with title and abstract scrutiny. ^b^ ARU: Anglia Ruskin University library search database.

The first level of screening focused on titles and abstracts based on face-value relevance to the authors and contributors. Literature based on differing medical professions and education as a general concept were excluded; however, consideration of inter-professional learning via simulation was included. A secondary screening excluded records based on content and relevance after a full-text review. An independent content review was conducted of the remaining studies, followed by discussions and consensus for inclusion. The purpose of the structured screening was to determine the most content-rich literature that would contribute to a broad and diverse narrative discussion of SBE for paramedics.

## Results

**Figure 1:**
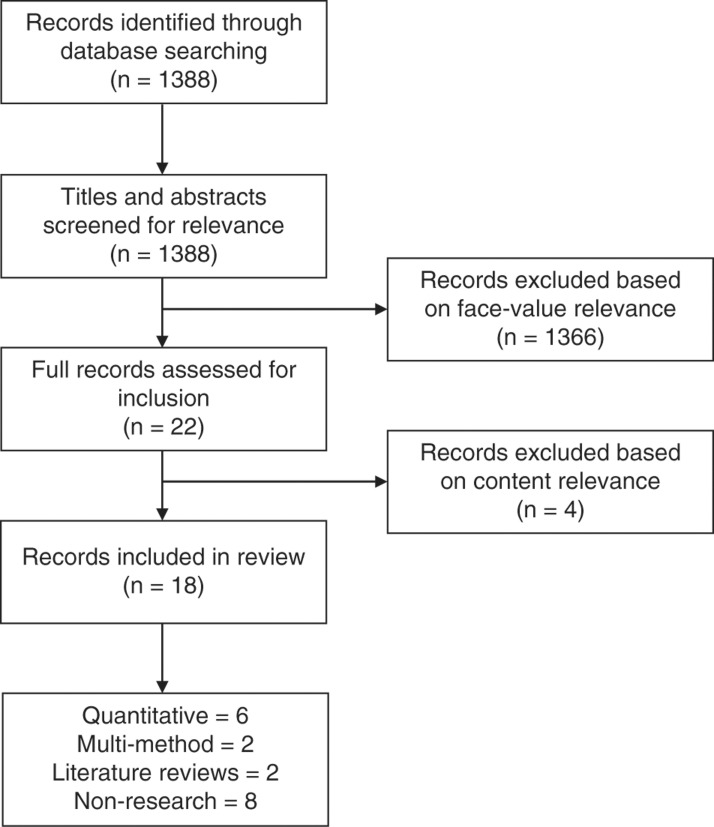
Figure 1. PRISMA flow diagram of the identification and screening process.

The original search yielded 1388 hits, with 22 records meeting the inclusion criteria based on face-value information during the first screening process. Following a secondary screening process assessing content and relevance, 18 records remained and were included in the analysis. Of the 18 papers included in this review, six used quantitative methods, two used multi-method approaches, two were literature reviews and eight were non-research papers. This gave a spread of healthcare and education approaches to the review, to give rounded representation of the literature. The screening process based on the PRISMA approach is shown in Figure 1.

### Data summary tables

Of the eight primary research studies included, two were conducted in the United Kingdom, three in the USA, two in Australia, one in Canada and two from other countries worldwide. Across all the literature, a range of concepts were discussed: Skill vs Scenario, Virtual Learning, Inter-Professional Learning, Fidelity, Cost, Equipment, Improvement of Competency, Patient Safety and Perception of Simulation. The characteristics of the literature were organised depending on the methodology of the study and are presented in Table 3, with texts from the literature used where possible to reflect an accurate account.

**Table 3.** Characteristics of literature included in the review.

*RESEARCH STUDIES*				
Author, year, title	Aim	Methods	Sample/population	Outcome/conclusion
**Alinier et al.** (2014) Immersive clinical simulation in undergraduate health care interprofessional education: Knowledge and perceptions	To explore the use of clinical simulation to enhance learning opportunities, inter-professional training and improved multi-professional working.	A quasi-randomised control group investigation using simulation sessions and questionnaires.	233 of 237 undergraduate students across various healthcare programmes from a British university: nursing, paramedic, radiography, physiotherapy and pharmacy. 116 experimental and 117 control.	The experimental group reported a higher perceived level of knowledge of other professions, were more confident working as part of a multidisciplinary team, had a greater appreciation of prequalification inter-professional learning opportunities and outscored on discipline knowledge.
**Birt et al.** (2017) Improving paramedic distance education through mobile mixed reality simulation	To explore the use of mobile mixed reality for distance learning of paramedic science interventions.	A design-based research with underlying action research mentality using user-supplied mobile phones and associated technologies to simulate direct laryngoscopy and foreign body removal.	137 of 159 2nd-year distance paramedic students across two rounds of the intervention from a university in Australia. 55 simulation and 82 control.	There is a statistically significant improvement for students who received the tools before residential school, both across the skill set and within individual skills.
**Butina et al.** (2013) Utilization of virtual learning environments in the allied health professions	To explore whether, and how, virtual learning environment instructional technology is being adapted in allied health education.	A descriptive study using an online survey with demographic and text responses to determine use, perceived pros and cons and utilisation outcomes.	42 of 126 academic leaders from member institutions of the Association of Schools of Allied Health Professions (ASAHP) in the USA.	Results show that 17 of the respondents use some form of virtual learning technology, and its use in other healthcare professions demonstrates the potential benefits to allied health education.
**Conradi et al.** (2009) Virtual patients in a virtual world: Training paramedic students for practice	To trial a replacement to traditional paper-based learning (PBL) with virtual patients (VPs).	An educational trial was conducted using a virtual world platform to conduct training scenarios, followed by student questionnaires and focus groups and facilitator reflections and interviews.	20 paramedic foundation degree students from two universities in the UK. 10 1st-year students from one, and 10 mixed 1st- and 2nd-year students from the other.	Feedback indicated that the virtual world platform engages students effectively in learning, despite some technology barriers, and students believe it could provide a more authentic learner environment than classroom-based PBL.
**Hall et al.** (2005) Human patient simulation is effective for teaching paramedic students endotracheal intubation	To determine whether the endotracheal intubation (ETI) success rate is different between paramedic students trained on a human patient simulator versus on human subjects in the operating room (OR).	A prospective, randomised, controlled trial using training on a patient simulator (10 hours) or human subjects (15 training intubations), followed by a measure of intubation success rate in the OR (15 intubations).	36 of 42 2nd-year paramedic students from an Institute of Technology in Canada. 18 simulator and 18 OR.	When tested in the OR, paramedic students who were trained in ETI on a simulator were as effective as students who trained on human subjects.
**McKenna et al.** (2015) Simulation Use in Paramedic Education Research (SUPER): A descriptive study	To characterise the use of simulation in initial paramedic education programmes to assist stakeholders’ efforts to target educational initiatives and resources.	A cross-sectional study using a census survey developed and revised using a consensus decision-making approach.	389 of 638 paramedic programmes either accredited by the Commission on Accreditation of Allied Health Education Programs (CAAHEP) or holding a letter of review in the USA.	Paramedic programmes have access to diverse simulation resources; however, faculty training and other programme resources appear to influence their use.
**Studnek et al.** (2011) The association between emergency medical services field performance assessed by high-fidelity simulation and the cognitive knowledge of practicing paramedics	To assess the association between the performance of practising paramedics on validated cognitive examination and their field performance.	An observational educational study using the cognitive portion of the national paramedic certification exam (NREMT) and a simulated EMS response assessment.	113 of 142 paramedics employed at an EMS agency in the USA.	Results demonstrated a significant association between a practising paramedic’s performance on a cognitive examination and their field performance in a simulated EMS response.
**Williams et al.** (2016) Simulation experiences of paramedic students: A cross-cultural examination	To compare simulation satisfaction among paramedic students.	A cross-sectional study using a paper-based English version of the Satisfaction with Simulation Experience Scale (SSES).	511 of 549 paramedic students from a university in Australia and a university in Jordan undertaking and undergraduate degree. 306 from Australia and 205 from Jordan.	This study demonstrated that simulation education is generally well received by students in Australia and Jordan, although Australian students reported having higher satisfaction levels than their Jordanian counterparts.
*LITERATURE REVIEWS*				
**Author, year, title**	**Aim**	**Methods**	**Review evidence**	**Outcome/conclusion**
**Abelsson et al.** (2014) Mapping the use of simulation in prehospital care – A literature review	To provide an overview of the development and foci of research on simulation in pre-hospital care practice.	An integrative literature review with a comprehensive overview of existing published research in the pre-hospital setting where interventions were carried out in a simulation context.	165 of 718 studies were included between 1984 and 2012 from across North America, Europe, Oceania, Asia and the Middle East. The main topics identified were Intubation, Trauma Care, Cardiac Pulmonary Resuscitation (CPR), Ventilation and Triage.	This review suggests that there are relatively few published articles focusing on simulation in pre-hospital healthcare. Simulation is described as a positive training and education method for pre-hospital medical staff. It provides opportunities to train in the assessment, treatment and implementation of procedures and devices under realistic conditions.
**Onan et al.** (2017) A review of simulation-enhanced, team-based cardiopulmonary resuscitation training for undergraduate students	To review and synthesise published studies that address the primary question: What are the features and effectiveness of educational interventions related to simulation-enhanced, team-based cardiopulmonary resuscitation training?	A systematic review of the medical literature to identify publications on the use of simulation-enhanced techniques for team-based resuscitation training, with a focus on their current and potential applications in cardiac arrest and emergency situations.	26 of 219 studies were included and evaluated against the modified Kirkpatrick’s 4-level model. The main topics identified were Satisfaction, Modification of attitudes/perceptions, Acquisition of knowledge/skills, Retention of knowledge/skills, Evidence of transfer of learning to clinical practice and Change in organisational practice.	The review concluded seven principles: Briefing, Resuscitation practice with feedback and reflection, Debriefing with feedback and reflection, Scenario and complexity, Teamwork, Refreshment and re-certification, Guidance and feedback tools; which were summarised under the main categories of effective planning, implementation, and evaluation of team training programmes specific to healthcare. Further studies need to focus on how simulation learning transfers to clinical practice and its effect on patient safety.
*NON-RESEARCH*				
**Author, year, title**	**Context**		**Conclusion/summary**	
**Boyle et al.** (2007) Contemporary simulation education for undergraduate paramedic students	An indoor simulation centre and an outdoor road trauma simulation centre provide a more realistic experience for undergraduate paramedic students managing a variety of clinical scenarios; at a university in Australia.		Clinical simulations are seen as a valid educational resource to improve and reduce pre-hospital errors via virtual trauma/medical simulated clinical scenarios. These innovative simulation centres will continue to facilitate contemporary clinical management principles, while utilising innovative strategies that allow interdisciplinary and multi-agency clinical learning opportunities for undergraduate education and practising paramedics.	
**Donaghy** (2016) Skills development at a paramedic accident simulation centre	An outdoor accident simulation centre offering pre- and post-registration paramedics the opportunity to experience a range of scenarios in a real-life but secure environment to apply theory and practice in complex situations; at a university in the UK.		Drawing on a sound theoretical base that embeds practical elements, the centre supports the concept of simulation and maintains a realistic approach to paramedic education and development. The ultimate aim of simulation, aside from offering students a diverse and realistic experience, is to improve patient safety and outcomes. Evidence suggests that improved patient outcomes follow simulation, and the centre strives to achieve this by applying theoretical evidence-based knowledge to practical work-based simulation teaching sessions.	
**Johnson & Patterson** (2006) Simulation education in emergency medical services for children	A descriptive piece exploring the current state of simulation education in emergency medical services, as derived from the aviation industry. It evaluates simulation utility and describes its application within emergency medicine.		Medical simulation has been utilised for over 20 years; however, it remains in its infancy. It remains an education technique that is expensive and labour intensive, and its true value has yet to be realised. Simulation can also be used to assess competencies in increasingly complex scenarios as learners progress through training. The future of simulation depends on the adoption of simulation-based competencies at all levels of training and across multiple disciplines.	
**Jones et al.** (2011) Simulation in prehospital care: Teaching, testing and fidelity	A descriptive piece exploring the imperative issues related to the psychological, environmental and equipment fidelity of simulation in the education of pre-hospital care personnel. It further explores the concept of fidelity as it relates to simulation education.		Debate around simulation principally focuses on high equipment fidelity; however, for many HEIs operating in times of austerity, this is a luxury they can ill afford. By moving away from the purely assessment-focused simulation experience, to a combination of strategies which include scenario and role play and continuous feedback techniques, a simulation environment may be created, enabling learning opportunities which focus on process not product.	
**Özkalp & Saygili** (2015) The effectiveness of similitor usage in the paramedic education	A descriptive piece exploring the use of educational simulation to present a learning environment that provides the possibility of a learner-centred experience rather than an experience where the patient is objective; and one that gives both confidence and support to the students, at universities in Turkey.		Sufficient knowledge and skills are needed in the emergency health services. Simulation-based health education is one of the best examples of experience-based learning. It enables the student to gain experience by making, repeating and learning from mistakes without any patient harm. It prepares them for thinking about their performance. A simulation environment aids in the transfer of learned skills to the clinical environment.	
**Peate** (2011) Using simulation to enhance safety, quality and education	A descriptive piece exploring the use of simulation as a teaching tool that allows healthcare workers to offer risk-free, safe and effective care, as well as enabling organisations to improve their systems of care and reduce cost.		Modelling and simulation have the potential to decrease healthcare error and costs. Safer paramedic practice is a realistic aspiration for all paramedics. Simulation offers an important route to safer care for patients, and this needs to be integrated more fully into all aspects of healthcare education. It can be used for the teaching of basic and advanced motor and interpersonal skills, using low- and high-fidelity simulation centres and equipment.	
**Power** (2011) Enhancing the student learning experience through interactive virtual reality simulation	A virtual simulation workshop was held as part of a project for the development and implementation of an innovative virtual simulation package. The aim was to enhance the student learning experience, expand students' range of clinically orientated cognitive skills and develop reflective practice and peer review; at a university in the UK.		The advantage of virtual environments is that there are limitless variations for the designers and teaching staff to create. However, from a teaching perspective, this methodology is quite labour intensive, requiring substantially greater use of resources in terms of time. Evaluation showed that the students enjoyed the workshop and felt it had been a very worthwhile learning experience.	
**Rice** (2013) The use of simulation mannequins in education	A descriptive piece exploring the use of mannequin-based simulation training for paramedics. It explores the fidelity and cost of mannequins and their effectiveness in emergency healthcare training.		Cognitive, social and personal resource non-technical skills are not immediately challenged in mannequin-based simulation without time and effort being given to the environment in which the mannequin is placed. Emerging literature suggests that mannequin-based simulation is a potentially valid method for delivering paramedic-specific education and training. However, any provider should consider carefully what they wish to address by purchasing mannequins for simulation.	

## Discussion

The review by McKenna et al. (2015), supported by data from Johnston and Batt (2019), confirms that the volume of published articles relating to simulation use for pre-hospital (paramedic) training is minimal. However, many prominent themes specific to paramedic education emerged from the literature. Simulation-Based Education (SBE) is used to describe a variety of educational practices in a range of settings, including the clinical assessment of practitioners, implementation of procedures and the use of simulated devices. The key emerging themes of this review are: Skill vs Scenario, Virtual Learning, Inter-professional Learning, Fidelity, Cost, Equipment, Improvement of Competency, Patient Safety and Perception of Simulation.

### Skill vs scenario

Teaching clinical skills is often perceived as the bedrock of simulation (Cook et al., 2011; Gunberg, 2012; McGaghie et al., 2010; Schaefer et al., 2011; Scholtz et al., 2013; Zendejas et al., 2013). For simple skill acquisition, basic trainers allow the development of psycho-motor processes without the associated cognitive stresses of a scenario.

Hall et al. (2005) compared human computerised simulation to real-life patients for the practice of endo-trachael intubation (ETI). Notably, this study challenges the construct that simulators are secondary in efficacy to true practice. Randomisation of 36 paramedic students with no prior ETI experience of either simulator or patient practice led to no difference when compared to 6 months’ practice. Simulator-trained students presented a greater first-pass success rate compared to the patient-trained group (84.4% / 80.0%, p = 0.27) (Hall et al., 2005). A successful intubation was defined as ‘correct tube placement within two attempts, determined by the anaesthesialogists’. They concluded that the use of simulation for teaching ETI is an effective adjunct, providing paramedic students with more opportunities to establish advanced airway management. However, simulation used specifically for ETI does not advocate removal of ‘true’ practice when establishing student competence based on infrequent practice and differing settings. The participants were under the direct supervision of experienced and competent colleagues, limiting the strength of the conclusion as the full cognitive load and responsibility were not entirely on the student.

Studnek et al. (2011) indicates that simulated scenario work provides the opportunity for full cognitive load and responsibility to be borne by the student(s). The opportunity afforded provides a higher-level thought process where known skills, knowledge or behaviour can be applied and modified to a given scenario (Studnek et al., 2011). Evidence suggests a high correlation between performance in cognitive examination and success with simulated patient encounters (Studnek et al., 2011). Extrapolation from this study shows simulation to identify areas of limited knowledge or for improvement. This should ideally be self-regulated. The maximal benefits of simulation depend heavily upon the student’s competence prior to entering the scenario. Simulation benefits include delivering patient-focused care, working in interdisciplinary teams and practising evidence-based medicine (Galloway, 2009). The facilitator or tutor’s role is pivotal, bridging the gap between the performance level and desired outcome with scenario-based simulation (Studnek et al., 2011).

### Virtual learning

Virtual learning is a new concept of the 21st century and allows students to learn away from the university environment. It has expanded substantially in higher education, and in recent years has been applied to medical courses.

Birt et al. (2017) used 3D printing alongside virtual reality to provide real environment experiences away from the university setting. This allowed students to practise advanced skills in a virtual reality distance environment prior to consolidation at university. Second-year paramedic science students were chosen as participants, with the only requirement being a specific mobile software (Birt et al., 2017). Results showed that students provided with the virtual simulation prior to practising at university had higher performance levels compared to those who did not (2.53 / 1.96; p = 0.031) (Birt et al., 2017). The authors concluded that virtual learning prior to university assessment is an effective way for students to increase competency levels in both overall performance and specific tasks (Birt et al., 2017). Autonomic skills can be ascertained through this method, but may have limited application due to substantial cost and time factors. A similar programme was adopted by Power (2011), which showed the concurrent use of virtual reality and simulation to consolidate students’ current knowledge and provide new opportunities to enhance further learning. This is through ‘real time’ scenarios being adhered to, limitless scenarios available to the students and important skills being reflected upon: communication, teamwork and patient management. This is supported by Conradi et al. (2009), which showed virtual reality-based Problem-Based Learning (PBL) provides students with the opportunity to immerse themselves in realistic patient scenarios. Second-year paramedic students were given clinical scenarios via interactive virtual reality to enhance their decision-making skills and ensure safe practice. Evidence suggests that patient engagement and clinical decision making is accelerated when using virtual patients rather than paper-based learning (Conradi et al., 2009; Power, 2011). The ability to test various skills via the use of simulation is appealing to both students and staff. However, virtual reality simulations are dependent on the software working consistently as well as compliance from the students. This method proves time-consuming for students to initially engage in and requires support from multiple services to ensure that it is used properly. Ideally, software that provides virtual reality simulations which is easy and effective to use will see the most improvement in paramedic learning (Conradi et al., 2009).

### Inter-professional learning

Paramedics often work alongside other emergency and healthcare professionals, and thus effective communication skills are essential. Simulation provides an environment for students to practise working alongside others in a scenario environment. With other professionals also using simulation, it provides a platform with which professions can learn about each other.

Alinier et al. (2014) used high-fidelity simulations in which students from a variety of healthcare courses took part and which focused on monitoring and reflecting on key skills such as communication, teamwork and collaboration. Students were split into non-randomised teams and briefed about each scenario (Alinier et al., 2014). Results showed an increase in confidence working with other professionals for those in the experiment group (3.27 / 2.99) and an increase in wanting further experience via simulation (4.35 / 4.02) (Alinier et al., 2014). Although there was not a significant increase in performance results, there was a consistent increase in inter-professional knowledge by the experimental group (Alinier et al., 2017). The study also highlighted the timing difficulty of coordinating students across the varied professions.

Onan et al. (2017) used simulation to reflect upon team-based CPR (Cardiopulmonary Resuscitation) and identified that it consolidates knowledge, rather than expanding it. Understandably, this is more difficult for individuals with less exposure to inter-professional learning. Methods such as written exams, multiple-choice questions and true/false questions were used to evaluate level of knowledge. The use of SBE allows sessions to be designed with debriefing and reflection as a core concept. This provides an environment which encourages students to work on areas of weakness (Onan et al., 2017). This is particularly effective for students lacking confidence (Rezmer et al., 2011). Evidence shows that teamwork is essential for CPR to be conducted effectively (Onan et al., 2017). Simulation allows for the development of teamwork through the provision of a variety of scenarios. It is important to reflect on learning styles and that simulation is not necessarily effective for all healthcare students when learning CPR. Therefore, SBE in inter-professional learning increases student satisfaction and acquisition of knowledge but does not necessarily improve competency levels (Onan et al., 2017).

### Fidelity

Fidelity in the healthcare environment is essential for translation to real-patient encounters. In line with the *Healthcare Simulation Dictionary*, fidelity means ‘the degree to which the simulation replicates the real event and/or workplace; this includes physical, psychological, and environmental elements’ (Lopreiato et al., 2020). SBE is consistent in providing students with relevant acquisition of knowledge and skills in the field of healthcare (Cook et al., 2011). SBE with high fidelity includes the actions of the student but also the equipment provided, and its psychological impact. It is common for simulation to face the issue of fidelity; however, when used as part of a larger student experience, simulation can be an advantageous option for preparing students.

Jones et al. (2011) states that simulation alone to provide students with knowledge, skills and experience is unreasonable and also near impossible. It is important to recognise that simulation is best used for consolidating knowledge and applying it practically (Jones et al., 2011). Evidence shows that pre-hospital high-fidelity equipment is lacking, primarily due to cost and inability to replicate life-like mannequins. This questions how effective simulation is for paramedic students as the lack of realism may affect real-patient treatment. Based on lack of high-fidelity equipment, high environmental fidelity increases in significance, as discussed by Donaghy (2016). The change in environment that the scenario is exploring is an integral aspect of learning for paramedic students, and this replication is crucial (Donaghy, 2016). Although at a high cost, re-creating different environments is more important to create ‘real’ scenarios (Donaghy, 2016). This identifies the importance of instructional design, in which the facilitators of scenarios can transfer knowledge and skills onto students, allowing them to have more preparation for the real world (Jones et al., 2011).

With a change in the role of paramedics, equipment fidelity has never been more crucial. Patient demographics in the pre-hospital setting are changing, in which critically unwell patients now make up the minority (AACE, 2011; Evans et al., 2014). Low-acuity patients cannot be easily replicated by high-fidelity mannequins; alternative options may be just as effective for learning (Rice, 2013). Prior use of these high-fidelity mannequins allowed for competency of skills to improve; however, it is reflective of old didactic paramedic education which focuses only on critically unwell patients (Rice, 2013). Therefore, the use of simulated patients may offer greater realism and deeper learning; this is dependent on the skills or knowledge desired.

### Cost

Simulation is a concept that has grown to become an expensive teaching method which encompasses complicated clinical elements of simulation that are limited to high-cost mannequins. For students to benefit the most from simulation, it is still believed that high-fidelity equipment and environments are needed and this has therefore become the focal point for simulation in paramedic education. Both studies discussed below are limited by omitting their economic analysis and providing costs of the simulation they researched.

Johnson and Patterson (2006) found that there are different modalities of simulation that target different aspects of training and thus result in different costs. For example, working on communication and patient-facing skills would only require an actor, or even just the educator, to fill this role (Johnson & Patterson, 2006). This was compared to practising Advanced Life Support (ALS), which requires mannequins that allow endotracheal intubation and cannulation and are substantially more expensive (Johnson & Patterson, 2006). Johnson and Patterson (2006) state that airway mannequins which can be used to practise advanced airways but not other advanced skills such as cannulation currently cost US$30,000. Pre-hospital use of mannequins for simulation is the common trend; however, they are not widely used due to their cost. Mannequins of the paediatric patient group are now becoming accessible and allow for paramedic education to specifically focus on treatment of these patients. Despite the cost, simulation provides paramedic students with the best opportunity to prepare and practise (Johnson & Patterson, 2006).

Peate (2011) indicates that the use of simulation has the potential to decrease healthcare costs altogether through the reduction of human error. Due to the expense of high-fidelity equipment and environments which provide students with the best learning opportunities, paramedic training differs greatly between organisations and pathways. This is an area which needs to be addressed and become consistent for the safest training of paramedics. The types of simulation available for educators should also be considered: computerised simulation vs. simulated patient vs. mannequin simulations (Peate, 2011). All provide different areas for paramedic students to learn from and thus need to be integrated together to provide the most effective training, which in turn reduces error and further healthcare costs in the real world (Peate, 2011). It is important to encourage the use of simulation for basic skills and knowledge rather than just focusing on advanced skills. This alongside focusing on teamwork and communication allows for simulation to be highly advantageous to paramedic training – and not necessarily at a high cost.

### Equipment

Emergency equipment, such as an intubation or cannulation kit, is used often throughout paramedic training and is one of the most effective ways to learn work-related psycho-motor skills. Being able to use their equipment will provide student paramedics with the most realistic experience of treating patients. The right type of equipment is essential when conducting simulations and is dependent on the nature of each scenario. By providing appropriate equipment, students can gain the necessary skills and knowledge in a safe environment.

Hall et al. (2005) showed that a Human Patient Simulator provides students with the same, if not better, experiences to practise endotracheal intubation. Higher success rates were found for students practising on a mannequin than for those practising on real patients, which suggests that learning opportunities using simulation are as effective as the real thing (Hall et al., 2005). The mannequins were also able to provide potential complications which could arise, and allow for safer practice and learning (Hall et al., 2005). This means students can correct any mistakes and build their confidence, with no detriment to the patient. Despite the high success rates of simulation, students who participated in the operating room with advanced clinicians on hand also had high success rates at endotracheal intubation (Hall et al., 2005). There is, however, more availability of simulation equipment than access to operating theatres and they can be used across all educational pathways.

Rice (2013) states that equipment for training paramedic students is essential and should therefore constantly be updated. This includes mannequins or replication of environments. He describes that high-fidelity equipment is not necessarily required for practising all skills and thus the equipment available should be appropriate (Rice, 2013). Rice (2013) constitutes high-fidelity mannequins as the ability to replicate physiological measurements, injuries or disease patterns representative of a multitude of clinical presentations. The purpose of simulation for students is to become competent clinicians and gain confidence – often coinciding with advanced skills. For example, student paramedics require more time carrying out endotracheal intubation and therefore advanced equipment is required (Rice, 2013). Therefore, equipment is highly effective for the practice of clinical skills, but it is important to note that key components of paramedic training should also include communication and teamwork – aspects that are not directly impacted by the equipment available. Similarly, it is important to recognise the changing demographics of patients presenting to paramedics; 9/10 emergency calls are deemed lower acuity, involving geriatrics and mental health patients (NHS England, 2018). With this in mind, the correct use of equipment is crucial but not necessarily the sole focus of paramedic training in the future.

### Improvement of competency

SBE provides the most effective environment for students to learn skills and gain knowledge until competent. All clinicians at differing levels, including student paramedics, will experience the four stages of competency: unconscious incompetence, conscious incompetence, conscious competence and unconscious competence (Sullivan and Wyatt, 2005). For student paramedics it is vital to become competent at clinical skills, and simulation is best suited to achieve this.

Williams et al. (2016) obtained data through a cross-sectional study showing that students felt the use of simulation allowed their confidence and skill levels to increase until they were able to reach a level of competency. Out of 511 students, 82.2% felt simulation was not a new learning technique despite a consistent change in year group (Williams et al., 2016). Data from both universities studied showed a mean score of 4.25 out of 5 for testing clinical abilities, although there was a difference in clinical reasoning skills and clinical decision making (4.36 / 3.51 and 4.37 / 3.59) (Williams et al., 2016). Each component was measured by the individual based on ‘The Satisfaction with Simulation Experience Scale’. This difference may reflect physical skills the students were able to carry out but may also reflect the differences in knowledge throughout the year groups. Similarly, the period of time that simulation has been accessible within the university impacts student’s feelings of competency. Those who have used simulation throughout all three years identify as more competent compared to those who are new to using simulation (Williams et al., 2016). Other factors may also have affected the results, including faculty development, time constraints, appropriateness and accuracy of the SBE design, which were not explored or excluded by the study. It is important that simulation is used to improve competency levels specific to the environment and skill required, ensuring students are satisfied and feel confident. The uses of simulation provide students with the opportunity to repetitively practise skills.

Competent clinicians are not only linked to skill performance, but also knowledge levels. Özkalp and Saygili (2015) found a significant association between paramedics who perform well in simulated environments and their cognitive levels (p = 0.02). Although there was a significant group who failed cognitive testing (20.6%), this is not necessarily reflective of how effective simulation is at improving competency levels (Özkalp and Saygili, 2015). Overall, Özkalp and Saygili (2015) found the use of simulation to be effective at improving physical skill level competency but not cognitive competency. Simulation is used in best practice to show new skills or refresh physical skills, and it therefore highlights clinicians’ weaknesses, allowing them to improve until competent. However, when using simulation to test clinical reasoning, evidence suggests that clinicians are less competent (Özkalp and Saygili, 2015). If other methods were used to test clinical reasoning, data may present differently and therefore simulation is not necessarily the best method for increasing cognitive competency. Simulation alongside other teaching methods would ensure best practice.

### Patient safety

Simulation is used for students to practise clinical skills until competent. Through lack of real patient availability and insufficient practice, SBE encourages students to become proficient in clinical skills with appropriate application. This in turn means human error is reduced on real patients, based on the high recall ratio and transferability of skills obtained (Özkalp and Saygili, 2015). The safety of patients is of upmost importance to clinicians and therefore all actions must be taken to ensure clinicians are safe. The practice of these skills to ensure patient safety has been historically difficult for paramedics to practise; however, simulation can now provide realistic scenarios that are more fitting.

Boyle et al. (2007) states that basic simulations are no longer effective at teaching student paramedics the skills and knowledge required to deal with the current patient population. Due to the vast change in the role of paramedics, simulation centres are now vital to provide high-fidelity environments best fit for paramedic practice (Boyle et al., 2007; Donaghy, 2016). The use of medical and trauma simulation centres exposes students to a variety of scenarios (Donaghy, 2016). SBE provides an environment to practise in and allows for mistakes to be made; this ensures that students learn from the experience (Boyle et al., 2007). Students are able to safely rehearse psycho-motor skills, teamwork and communication skills where there is no detrimental impact on patients (Boyle et al., 2007). Simulation centres through the use of immersive simulation are effective for advanced skills, but basic skill acquisition requires low-fidelity environments (Peate, 2011). Donaghy (2016) states that simulation centres, although effective for undergraduate students, provide less ideal simulations for master’s students and are unsurprisingly expensive to set up and run. SBE provides the opportunity for debriefs to be utilised and is effective at reducing errors before actual patient contact. Dependent on the design of the simulation, reflection can contribute to optimising learning opportunities. The use of simulation centres limits itself to focusing on trauma; paramedics face limited exposure to trauma and therefore simulation centres providing medical cases are also required (Boyle et al., 2017; Donaghy, 2016).

Peate (2011) states that simulation is inherently useful at creating safer care for patients and therefore should be integrated throughout all paramedic education pathways. The vast complexity of simulation allows for numerous opportunities to arise and is no longer limited to expense or time (Peate, 2011). This is supported by Alinier et al. (2014), who found that the use of immersive simulations meant students were more confident and competent at appropriately treating patients. Thorough use of simulation has the potential for students to fall into ‘habit’ when faced with difficult scenarios and may reduce human error (Peate, 2011). Although not applicable to all patient presentations, this nature of ‘habit’ can result in successful patient outcomes – particularly for trauma patients (Peate, 2011). Simulation provides opportunities not only to gain physical skill level competency, but also to work in teams and focus on communication; all human error factors which, if ineffective, can be detrimental to the patient (Peate, 2011). To increase patient safety, interactive simulations are best as they allow students to commit, understand and see the implications of their actions.

### Perceptions of simulation

Perceptions of simulation vary greatly. The experience of the learner can make a substantive difference to the effectiveness of simulation. A dichotomy of engagement exists where mature students are often more receptive to simulation. As there is a variety of types of simulation, perceptions may differ accordingly and can be representative of prior skill level/knowledge and aspirations from the simulation.

McKenna et al. (2015) discusses that the term ‘simulation’ has a multitude of definitions and is therefore difficult to categorise into one. Since this publication, the globally recognised document *The Healthcare Simulation Dictionary* has generated the definition for ‘simulation’. Simulation is used for numerous educational reasons, emphasising the easiness of differing perceptions. Some believe simulation is most effective at teaching student paramedics the required skills and knowledge until competent and is a good substitute for selective clinical experiences (Cook et al., 2011; Dickison, 2010; McKenna et al., 2015; Simon et al., 2012). However, when actually applied to pre-hospital students, simulation is believed not to be a sufficient replacement for clinical experience (McKenna et al., 2015). This is based on the challenging environments and scenarios which cannot be experienced by simulation. However, recent research shows the ability of simulation to provide these conditions, which are comparable to the clinical setting. As these are just as effective, they can be an appropriate adjunct (Hayden et al., 2014; Lapkin et al., 2010; Meyers et al., 2011; Sportsman et al., 2011). Different structures in pedagogy, different experiences or knowledge levels can all effect the perceptions of simulations and their effectiveness when provided to students (McKenna et al., 2015)

Simulation no longer only consists of mannequins and physical scenarios, but now encompasses a virtual learning aspect as well. Butina et al. (2013) found, unsurprisingly, that the use of virtual learning for simulations was not consistent across all educational providers. This is reflective of the perceptions being identified from higher management rather than from the students themselves (Butina et al., 2013). Although 40.7% of the educators state that they do use virtual learning programmes, only 17% of these use the same definition of virtual learning as the researchers (Butina et al., 2013). This shows the vast differences in definitions and applications of simulation, with concurrent use of virtual reality, when applied to paramedic education (Butina et al., 2013). Future work into simulation is required to provide accurate consistency among all pathways of paramedic education, including the undergraduate degree or apprenticeship route through the ambulance service.

### Limitations of simulation

The use of SBE does have some limitations. Firstly, high-fidelity equipment and high-fidelity environments are expensive, despite providing ideal opportunities for realistic learning opportunities of both skill and knowledge acquisition. The expense of high-fidelity simulations is not easily available to all educational providers, often requiring grants, and therefore there is a difference in teaching methods and results. This, alongside the differing views on what simulation is, has resulted in vast differences in the use and application of simulation. Simulation is not necessarily used effectively by all educators and may see inconsistent results in the level of skilled, knowledgeable paramedics created, particularly for specialised roles.

## Review limitations

There are some limitations to this review. This is a scoping review and is intended only to map and highlight literature descriptively in the area of simulation as applied to paramedic education, and it does not involve the same critical evaluation as a systematic review to answer a specific research question. The authors recognise that there may be many advances in simulation technology and practice; however, it remains clear from our search strategy that there is a paucity of literature published on this topic. The review is heavily influenced by the majority of quantitative research, with little exploration of the qualitative nature of simulation. Identifying and analysing qualitative characteristics of simulation, such as high stress level features, will allow for adaptation of simulation to best tackle these components. Further research is required to specifically identify the uses and applicability of simulation-based education for paramedics in modern times and to look into alternative means that are accessible to all providers of education.

## Conclusion

It is evident that SBE is a primary teaching modality, reliably used to educate and train paramedics. Simulation is inherently effective at teaching clinician’s skills and building student competence in particular areas. Similarly, it is effective at providing paramedics with the experiences and opportunities to learn in varied environments using differing techniques, including multi-professional teams, immersive simulation or simulated environments. This allows students to be able to apply the relevant skills and knowledge when faced with real patients. Exploring further research into the effectiveness of SBE for student paramedics will identify how appropriate simulation is as a teaching modality and how it can be expanded in modern times. It is vital to understand the perspectives and the perception of educators and students to inform these adaptations to simulation. Consistency between education providers in their use of SBE is imperative to ensure that student paramedics gain valuable knowledge and skills to treat real-life patients.

## Acknowledgements

The authors wish to acknowledge the contributions of Oliver Smith and Molly Tarawally who assisted in initial draft searching and paper screening.

## Author contributions

ED initiated and conceptualised the review, compiled search strategies, provided paper screening and wrote the manuscript. BW, a paramedic student, attained an undergraduate research internship to assist with the review. BW conducted the database searches, and assisted with secondary screening and the writing of the manuscript. ED acts as the guarantor for this article.

## Conflict of interest

None declared.

## Ethics

Not required.

## Funding

There were no direct funds received. Anglia Ruskin University supported the review through the Emergency Medicine Research Group, by providing library access and academic support. BW, a paramedic student, received an undergraduate research internship worth £2000 to assist with the review.
